# *catena*-Poly[[diaqua­bis­(isoquinoline-κ*N*)cobalt(II)]-μ-succinato-κ^2^ *O* ^1^:*O* ^4^]

**DOI:** 10.1107/S1600536810023895

**Published:** 2010-06-26

**Authors:** Meng-Jiao Li, Jing-Jing Nie, Duan-Jun Xu

**Affiliations:** a Department of Chemistry, Zhejiang University, People’s Republic of China

## Abstract

In the title compound, [Co(C_4_H_4_O_4_)(C_9_H_7_N)_2_(H_2_O)_2_]_*n*_, the Co^II^ cation, located on an inversion center, is coordinated by two succinate anions, two isoquinoline ligands and two water mol­ecules in a distorted octa­hedral geometry. The succinate anion, located across another inversion center, bridges the Co cations, forming polymeric chains running along the *b* axis. The partially overlapped arrangement of parallel isoquinoline ring systems of adjacent polymeric chains and the shorter face-to-face distance of 3.402 (6) Å indicates the existence of weak π–π stacking in the crystal structure. Classical intra- and inter­molecular O—H⋯O hydrogen bonding and weak non-classical inter­molecular C—H⋯O hydrogen bonding help to stabilize the crystal structure.

## Related literature

For general background to π–π stacking, see: Deisenhofer & Michel (1989 ▶); Su & Xu (2004 ▶); Xu *et al.* (2007 ▶). For two related isoquinoline complexes, see: Li *et al.* (2009*a* ▶,*b* ▶). For a related polymeric Ni^II^ complex bridged by succinate anions, see: Liu *et al.* (2003 ▶).
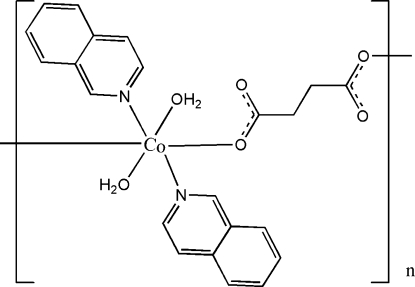


## Experimental

### 

#### Crystal data

[Co(C_4_H_4_O_4_)(C_9_H_7_N)_2_(H_2_O)_2_]
*M* *_r_* = 469.35
Monoclinic, 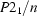

*a* = 11.258 (4) Å
*b* = 9.023 (5) Å
*c* = 11.390 (7) Å
β = 114.667 (5)°
*V* = 1051.4 (9) Å^3^
*Z* = 2
Mo *K*α radiation
μ = 0.86 mm^−1^
*T* = 294 K
0.24 × 0.14 × 0.12 mm

#### Data collection

Rigaku R-AXIS RAPID IP diffractometer
Absorption correction: multi-scan (*ABSCOR*; Higashi, 1995 ▶) *T*_min_ = 0.788, *T*_max_ = 0.862
4907 measured reflections
1891 independent reflections
1165 reflections with *I* > 2σ(*I*)
*R*_int_ = 0.040

#### Refinement

*R*[*F* ^2^ > 2σ(*F* ^2^)] = 0.033
*wR*(*F* ^2^) = 0.057
*S* = 0.82
1891 reflections
142 parameters
H-atom parameters constrained
Δρ_max_ = 0.23 e Å^−3^
Δρ_min_ = −0.23 e Å^−3^

### 

Data collection: *PROCESS-AUTO* (Rigaku, 1998 ▶); cell refinement: *PROCESS-AUTO*; data reduction: *CrystalStructure* (Rigaku/MSC, 2002 ▶); program(s) used to solve structure: *SIR92* (Altomare *et al.*, 1993 ▶); program(s) used to refine structure: *SHELXL97* (Sheldrick, 2008 ▶); molecular graphics: *ORTEP-3* (Farrugia, 1997 ▶); software used to prepare material for publication: *WinGX* (Farrugia, 1999 ▶).

## Supplementary Material

Crystal structure: contains datablocks I, global. DOI: 10.1107/S1600536810023895/rk2211sup1.cif

Structure factors: contains datablocks I. DOI: 10.1107/S1600536810023895/rk2211Isup2.hkl

Additional supplementary materials:  crystallographic information; 3D view; checkCIF report

## Figures and Tables

**Table 1:** Hydrogen-bond geometry (Å, °)

*D*—H⋯*A*	*D*—H	H⋯*A*	*D*⋯*A*	*D*—H⋯*A*
O1*W*—H1*A*⋯O2^i^	0.90	1.89	2.774 (3)	169
O1*W*—H1*B*⋯O2	0.87	1.90	2.689 (3)	150
C5—H5⋯O2^ii^	0.93	2.56	3.487 (5)	176

Symmetry codes: (i) 
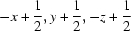
; (ii) 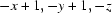.

**Fig. 1.**
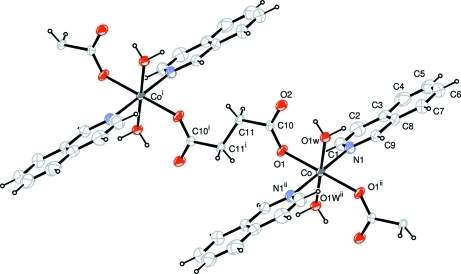
A part of the polymeric molecular structure of the title compound with atom numbering scheme. Displacement ellipsoids are drawn at 50% probability level. H atoms are presented as a small spheres of arbitrary radius. Symmetry codes: (i) 1-x, -y, 1-z; (ii) 1-x, 1-y, 1-z.

## supplementary crystallographic information

### Comment

The π···π stacking between aromatic rings is an important non-covalent interaction and correlated with the electron transfer process in some biological systems (Deisenhofer & Michel, 1989). As part of our ongoing investigation on the nature of π···π stacking (Su & Xu, 2004; Xu *et al.*, 2007), the title complex incorporating isoquinoline ligand has recently been prepared in the laboratory and its crystal structure is reported here.

A part of the polymeric molecular structure is shown in Fig. 1. The Co^II^ cation located on an inversion center is coordinated by two succinate anions, two isoqiunoline ligands and two water moleculaes with a distorted octahedral geometry. The succinate anion is located across another inversion center, and bridges Co cations to form the one-dimensional polymeric chains running along the crystallographic *b* axis, similar to that found in a Ni^II^ complex bridged by siccinate anions (Liu *et al.*, 2003). The carboxyl group is oriented with respect to the carbon skeleton of succinate anion at a dihedral angle of 28.4 (2)°.

The partially overlapped arrangement of parallel isoqiunoline ring systems of adjacent polymeric chains related by a symmetry operation of (1-*x*, 1-*y*, -*z*) and shorter face-to-face distance of 3.402 (6)Å indicate the existence of weak π···π stacking in the crystal structure. Classical intra- and intermolecualr O–H···O hydrogen bonding and weak non-classical intermolecular C–H···O hydrogen bonding help to stabilize the crystal structure (Table 1).

### Experimental

The CoCl_2_^.^6H_2_O (0.48 g, 2 mmol), succinic acid (0.24 g, 2 mmol), NaOH (0.16 g, 4 mmol) and isoquinoline (0.23 ml, 2 mmol) were dissolved in a water/ethanol solution (20 ml, 1:1). The solution was refluxed for 4 h. The reaction mixture was cooled to room temperature and filtered. The single crystals were obtained from the filtrate after two weeks.

### Refinement

Water H atoms were located in a difference Fourier map and refined as-riding in as-found relative positions with *U*_iso_(H) = 1.2*U*_eq_(O). Other H atoms were placed in calculated positions with C–H = 0.93Å (aromatic) and 0.97Å (methylene), and refined in riding mode with *U*_iso_(H) = 1.2*U*_eq_(C).

### Crystal data

**Table:**

[Co(C_4_H_4_O_4_)(C_9_H_7_N)_2_(H_2_O)_2_]	*F*(000) = 486
*M**_r_* = 469.35	*D*_x_ = 1.482 Mg m^−^^3^
Monoclinic, *P*2_1_/*n*	Mo *K*α radiation, λ = 0.71073 Å
Hall symbol: -P 2yn	Cell parameters from 2408 reflections
*a* = 11.258 (4) Å	θ = 3.5–24.6°
*b* = 9.023 (5) Å	µ = 0.86 mm^−^^1^
*c* = 11.390 (7) Å	*T* = 294 K
β = 114.667 (5)°	Prism, pink
*V* = 1051.4 (9) Å^3^	0.24 × 0.14 × 0.12 mm
*Z* = 2	

### Data collection

**Table:**

Rigaku R-AXIS RAPID IP diffractometer	1891 independent reflections
Radiation source: fine-focus sealed tube	1165 reflections with *I* > 2σ(*I*)
graphite	*R*_int_ = 0.040
Detector resolution: 10.0 pixels mm^-1^	θ_max_ = 25.2°, θ_min_ = 3.3°
ω–scan	*h* = −13→13
Absorption correction: multi-scan (*ABSCOR*; Higashi, 1995)	*k* = −10→8
*T*_min_ = 0.788, *T*_max_ = 0.862	*l* = −13→13
4907 measured reflections	

### Refinement

**Table:**

Refinement on *F*^2^	Primary atom site location: structure-invariant direct methods
Least-squares matrix: full	Secondary atom site location: difference Fourier map
*R*[*F*^2^ > 2σ(*F*^2^)] = 0.033	Hydrogen site location: inferred from neighbouring sites
*wR*(*F*^2^) = 0.057	H-atom parameters constrained
*S* = 0.82	*w* = 1/[σ^2^(*F*_o_^2^) + (0.0207*P*)^2^] where *P* = (*F*_o_^2^ + 2*F*_c_^2^)/3
1891 reflections	(Δ/σ)_max_ < 0.001
142 parameters	Δρ_max_ = 0.23 e Å^−^^3^
0 restraints	Δρ_min_ = −0.23 e Å^−^^3^

### Special details

**Table:**

Geometry. All s.u.'s (except the s.u. in the dihedral angle between two l.s. planes) are estimated using the full covariance matrix. The cell s.u.'s are taken into account individually in the estimation of s.u.'s in distances, angles and torsion angles; correlations between s.u.'s in cell parameters are only used when they are defined by crystal symmetry. An approximate (isotropic) treatment of cell s.u.'s is used for estimating s.u.'s involving l.s. planes.
Refinement. Refinement of *F*^2^ against ALL reflections. The weighted *R*-factor *wR* and goodness of fit *S* are based on *F*^2^, conventional *R*-factors *R* are based on *F*, with *F* set to zero for negative *F*^2^. The threshold expression of *F*^2^ > σ(*F*^2^) is used only for calculating *R*-factors(gt) *etc*. and is not relevant to the choice of reflections for refinement. *R*-factors based on *F*^2^ are statistically about twice as large as those based on *F*, and *R*-factors based on ALL data will be even larger.

### Fractional atomic coordinates and isotropic or equivalent isotropic displacement parameters (Å^2^)

**Table:**

	*x*	*y*	*z*	*U*_iso_*/*U*_eq_	
Co	0.5000	0.5000	0.5000	0.03143 (14)	
N1	0.5562 (2)	0.5432 (2)	0.34392 (16)	0.0394 (6)	
O1	0.50790 (18)	0.27225 (15)	0.47973 (14)	0.0392 (4)	
O2	0.31969 (17)	0.21732 (17)	0.31456 (15)	0.0434 (5)	
O1W	0.29833 (13)	0.50231 (19)	0.37221 (11)	0.0369 (4)	
H1A	0.2673	0.5677	0.3070	0.044*	
H1B	0.2846	0.4213	0.3263	0.044*	
C1	0.6531 (3)	0.4608 (3)	0.3341 (2)	0.0513 (8)	
H1	0.6940	0.3889	0.3965	0.062*	
C2	0.6927 (3)	0.4788 (4)	0.2376 (2)	0.0572 (8)	
H2	0.7600	0.4205	0.2357	0.069*	
C3	0.6326 (3)	0.5844 (3)	0.1411 (2)	0.0462 (7)	
C4	0.6658 (3)	0.6069 (4)	0.0350 (3)	0.0647 (9)	
H4	0.7314	0.5506	0.0274	0.078*	
C5	0.6012 (4)	0.7107 (4)	−0.0549 (3)	0.0698 (10)	
H5	0.6228	0.7246	−0.1246	0.084*	
C6	0.5030 (4)	0.7973 (4)	−0.0451 (3)	0.0700 (10)	
H6	0.4603	0.8681	−0.1080	0.084*	
C7	0.4689 (3)	0.7791 (3)	0.0560 (2)	0.0583 (9)	
H7	0.4034	0.8372	0.0620	0.070*	
C8	0.5334 (3)	0.6720 (3)	0.1506 (2)	0.0410 (7)	
C9	0.4999 (3)	0.6448 (3)	0.2554 (2)	0.0399 (6)	
H9	0.4343	0.7022	0.2620	0.048*	
C10	0.4249 (2)	0.1817 (2)	0.4066 (2)	0.0294 (6)	
C11	0.4568 (2)	0.0192 (2)	0.43056 (17)	0.0335 (6)	
H11A	0.4999	−0.0128	0.3768	0.040*	
H11B	0.3758	−0.0360	0.4039	0.040*	

### Atomic displacement parameters (Å^2^)

**Table:**

	*U*^11^	*U*^22^	*U*^33^	*U*^12^	*U*^13^	*U*^23^
Co	0.0357 (3)	0.0151 (2)	0.0285 (2)	−0.0009 (3)	−0.00145 (17)	0.0019 (2)
N1	0.0390 (13)	0.0305 (13)	0.0367 (11)	0.0033 (10)	0.0040 (9)	0.0038 (9)
O1	0.0391 (11)	0.0153 (8)	0.0426 (10)	−0.0025 (9)	−0.0035 (8)	−0.0015 (8)
O2	0.0444 (12)	0.0209 (10)	0.0399 (9)	0.0028 (8)	−0.0073 (8)	−0.0005 (8)
O1W	0.0410 (9)	0.0205 (8)	0.0306 (7)	0.0015 (10)	−0.0034 (6)	0.0011 (8)
C1	0.0472 (19)	0.044 (2)	0.0487 (15)	0.0098 (15)	0.0061 (13)	0.0064 (13)
C2	0.0420 (17)	0.059 (2)	0.0639 (17)	0.0070 (17)	0.0159 (14)	−0.0038 (17)
C3	0.0436 (19)	0.0442 (18)	0.0443 (15)	−0.0146 (15)	0.0119 (13)	−0.0090 (14)
C4	0.061 (2)	0.073 (2)	0.0654 (19)	−0.028 (2)	0.0313 (18)	−0.0158 (19)
C5	0.086 (3)	0.070 (3)	0.056 (2)	−0.041 (2)	0.033 (2)	−0.0074 (19)
C6	0.096 (3)	0.056 (2)	0.0468 (18)	−0.020 (2)	0.0196 (19)	0.0072 (16)
C7	0.072 (2)	0.0434 (19)	0.0503 (18)	−0.0043 (17)	0.0166 (17)	0.0055 (15)
C8	0.0474 (19)	0.0300 (15)	0.0382 (14)	−0.0077 (14)	0.0104 (12)	0.0015 (13)
C9	0.0428 (17)	0.0304 (15)	0.0383 (14)	−0.0012 (13)	0.0087 (12)	0.0010 (13)
C10	0.0384 (16)	0.0185 (13)	0.0264 (11)	0.0001 (13)	0.0087 (11)	0.0025 (11)
C11	0.0413 (14)	0.0142 (13)	0.0323 (11)	0.0007 (12)	0.0030 (9)	0.0003 (11)

### Geometric parameters (Å, °)

**Table:**

Co—N1^i^	2.157 (2)	C3—C8	1.409 (4)
Co—N1	2.157 (2)	C3—C4	1.420 (4)
Co—O1	2.0740 (18)	C4—C5	1.354 (4)
Co—O1^i^	2.0740 (18)	C4—H4	0.9300
Co—O1W^i^	2.1249 (14)	C5—C6	1.397 (5)
Co—O1W	2.1249 (14)	C5—H5	0.9300
N1—C9	1.314 (3)	C6—C7	1.367 (4)
N1—C1	1.363 (3)	C6—H6	0.9300
O1—C10	1.259 (3)	C7—C8	1.404 (4)
O2—C10	1.253 (3)	C7—H7	0.9300
O1W—H1A	0.8975	C8—C9	1.416 (3)
O1W—H1B	0.8743	C9—H9	0.9300
C1—C2	1.357 (3)	C10—C11	1.507 (3)
C1—H1	0.9300	C11—C11^ii^	1.511 (4)
C2—C3	1.398 (4)	C11—H11A	0.9700
C2—H2	0.9300	C11—H11B	0.9700
			
O1—Co—O1^i^	180.0	C2—C3—C4	123.6 (3)
O1—Co—O1W^i^	89.04 (7)	C8—C3—C4	118.9 (3)
O1^i^—Co—O1W^i^	90.96 (7)	C5—C4—C3	119.6 (3)
O1—Co—O1W	90.96 (7)	C5—C4—H4	120.2
O1^i^—Co—O1W	89.04 (7)	C3—C4—H4	120.2
O1W^i^—Co—O1W	180.0	C4—C5—C6	121.3 (3)
O1—Co—N1^i^	87.33 (7)	C4—C5—H5	119.4
O1^i^—Co—N1^i^	92.67 (7)	C6—C5—H5	119.4
O1W^i^—Co—N1^i^	91.76 (7)	C7—C6—C5	120.7 (3)
O1W—Co—N1^i^	88.24 (7)	C7—C6—H6	119.7
O1—Co—N1	92.67 (7)	C5—C6—H6	119.7
O1^i^—Co—N1	87.33 (7)	C6—C7—C8	119.5 (3)
O1W^i^—Co—N1	88.24 (7)	C6—C7—H7	120.3
O1W—Co—N1	91.76 (7)	C8—C7—H7	120.3
N1^i^—Co—N1	180.0	C7—C8—C3	120.0 (2)
C9—N1—C1	117.7 (2)	C7—C8—C9	122.1 (3)
C9—N1—Co	123.03 (19)	C3—C8—C9	117.8 (2)
C1—N1—Co	119.26 (16)	N1—C9—C8	123.7 (3)
C10—O1—Co	131.47 (16)	N1—C9—H9	118.2
Co—O1W—H1A	120.6	C8—C9—H9	118.2
Co—O1W—H1B	105.9	O2—C10—O1	124.7 (2)
H1A—O1W—H1B	98.3	O2—C10—C11	118.2 (2)
C2—C1—N1	123.1 (2)	O1—C10—C11	117.2 (2)
C2—C1—H1	118.5	C10—C11—C11^ii^	114.4 (2)
N1—C1—H1	118.5	C10—C11—H11A	108.7
C1—C2—C3	120.3 (3)	C11^ii^—C11—H11A	108.7
C1—C2—H2	119.9	C10—C11—H11B	108.7
C3—C2—H2	119.9	C11^ii^—C11—H11B	108.7
C2—C3—C8	117.4 (2)	H11A—C11—H11B	107.6

Symmetry codes: (i) −*x*+1, −*y*+1, −*z*+1; (ii) −*x*+1, −*y*, −*z*+1.

### Hydrogen-bond geometry (Å, °)

**Table:**

*D*—H···*A*	*D*—H	H···*A*	*D*···*A*	*D*—H···*A*
O1W—H1A···O2^iii^	0.90	1.89	2.774 (3)	169
O1W—H1B···O2	0.87	1.90	2.689 (3)	150
C5—H5···O2^iv^	0.93	2.56	3.487 (5)	176

Symmetry codes: (iii) −*x*+1/2, *y*+1/2, −*z*+1/2; (iv) −*x*+1, −*y*+1, −*z*.
